# Editorial: The 11^**th**^ Edition of the International Meeting of the SPCE-TC: Advances in Stem Cells and Cell Therapies

**DOI:** 10.3389/fcell.2021.720554

**Published:** 2021-07-14

**Authors:** Joana P. Miranda, Susana Solá

**Affiliations:** Faculty of Pharmacy, Research Institute for Medicines (iMed.ULisboa), Universidade de Lisboa, Lisbon, Portugal

**Keywords:** stem cell repair mechanisms, neuroscience, cancer, tissue engineering, cell development, hepatology

The key role of stem cells on tissue development, maintenance, and repair is becoming increasingly evident in health and disease. The special issue on *The 11*^*th*^ *Edition of the International Meeting of the SPCE-TC: Advances in Stem Cells and Cell Therapies* gathers several scientific contributions in this area while featuring the collaborative and interdisciplinary approach among Portuguese scientists within the stem cell community.

With the main focus on stem cells and regenerative medicine, our selected content embraces novel discoveries on metabolic and cell reprogramming, tissue engineering, regenerative medicine and cell transplantation while highlighting major advances in developmental biology, cancer and neuroscience scientific fields.

Original research articles include novel stem cell engineering approaches to breakthrough neuroscience. For example, Rocha et al. reveal that the combination of two type of cells in 3D biodegradable hydrogels promotes an efficient re-vascularization in trauma-related injuries of the central nervous system. In fact, acute traumatic spinal cord injury is a devastating event without effective therapeutic approach (Ahuja et al., 2017). In this study, the authors show that, under specific bio-engineering conditions, adipose-derived stem cells are capable of regulating protein expression in human umbilical vein endothelial cells to stimulate neuritogenesis and increase vascularization of dorsal root ganglia explants. These data open up promising avenues toward the implantion of this biomaterial-based cell therapy after spinal cord injury, possibly inducing revascularization and functional recovery. Regarding the value of neural organoids for disease modeling and therapy, Gomes et al. have managed to derive both dorsal and ventral 3D structures from health individuals- and Rett patient-specific induced pluripotent stem cells (hiPSCs) and study the organization and functional network complexity of the Rett Syndrome. Although brain organoids derived from Rett patients have been already used to identify promising treatments (Samarasinghe et al., 2019; Trujillo et al., 2021) this work provides a deeper understanding of the neural defects associated early stages of the developmental process in this Syndrome. Noteworthy, a premature development of the deep-cortical layer and a lower expression of neural progenitor cells were observed in dorsal organoids of female Rett-derived organoids, along with impairments of interneuron's migration and other electrophysiological and functional defects. In line with the previous works, Serras et al. further demonstrate the value of tissue organoids, in particular, 3D liver models for drug development. Around 40–50% of the drug candidates associated with hepatotoxicity in humans do not present the same toxicological concern in animal models (van Tonder et al., 2013). This has led to the proposal that the better the quality of non-clinical safety profiles, the higher the success rates for moving phase II upward (Cook et al., 2014; Walker et al., 2020). Consequently, *in vitro* liver models are growing strong while new drugs advance into clinical trials. This comprehensive review addresses the drug-induced hepatotoxicity mechanisms and the currently available 3D liver *in vitro* models, their characteristics, as well as their advantages and limitations for human hepatotoxicity assessment.

In addition, and going back to neuroscience field, the use of hiPSC to dissect neuropathological mechanisms was also performed by Lopes et al., who reveal profound alterations on mitochondrial biogenesis, function and morphology in both iPSC and neural stem cells derived from Huntington disease (HD) patients. HD is caused by CAG repeat expansion in the HTT gene and, like other neurodegenerative conditions, this disease continues to lack an effective cure. In this work, the authors demonstrate that deletion of CAG repeat by CRISPR/Cas9 technology ameliorates all mitochondrial phenotypes of HD-derived cells, bringing significant new information to tackle metabolic dysfunction associated with this neurological condition.

The remarkable contribution of Garcez et al. is another step toward our understanding on the role of mitochondrial dynamics and morphology in stem cell activity. Here, it has been elegantly revealed that mitochondrial dynamics are pivotal to assure basal oxidative phosphorylation levels in female germ stem cells of Drosophila ovary, and, importantly, that the number and morphology of these cells is greatly dependent on their basal respiration levels. Indeed, these findings are absolutely in line with recent studies demonstrating that aging-induced germline stem cell loss is dependent on mitochondrial dynamic shifts (Amartuvshin et al., 2020) and that maintenance of male germline stem cells relies on mitochondrial fusion processes (Demarco et al., 2019).

Still in the developmental biology field, this Research Topic also includes two personal perspectives from Seco et al. and from Soares-da-Silva et al. Specifically, Seco et al. discuss the embryonic source of aortic hematopoietic stem cells, a matter of intense debate within in the hematopoietic development field. The authors reflect on recent fate-mapping discoveries to re-interpretate classical studies in avian embryos and unify principles that better clarify the identity and origin of aortic hematopoietic stem cells. They also present conflicted data and discuss how future research may contribute to clarify some controversies, while also providing exciting data in chick embryos. On the other hand, Soares-da-Silva et al. explore hematopoietic and hepatic fetal systems concurrent establishment and evaluate to what degree they modulate their respective development. Indeed, deeper insights on the dynamics of fetal liver composition along development, and on how these different cell types impact hematopoiesis, are needed (Ema and Nakauchi, 2000). As insights into the molecular networks governing physiological hematopoietic stem cell (HSC) expansion accumulate, it is foreseeable that strategies to enhance HSC proliferation will be also improved.

Moving to cancer biology, Boemi et al. reveal for the first time that tamoxifen has no significant effect on cellular functions of adipose-derived stem cells. This ex-vivo single-center study come to contradict previous *in vitro* reports showing that tamoxifen inhibits proliferation and multi-lineage differentiation rates of adipose-derived stem cells (Pike et al., 2015), rising the discussion in this area of research. Further insights on the fast-growing cancer stem cells research area can also be found on our review collection, in which Pádua et al. emphasize the relevance of several transcription factors as potential biomarkers for cancer stem cells, but also as putative therapeutic targets in gastric and colorectal cancer. Still within the cancer field, but with a different approach, Silva M. et al., hold a promising future for the use of human mesenchymal stromal cells (MSCs), as cell delivery systems for anticancer proteins, due to their unique biological features (Ayuzawa et al., 2009). Upon treatment with hazurin-MSC-secretome, the authors observed a decrease in cancer cell proliferation, migration, and invasion, as well as an increase in cell death of lung and breast cancer cell lines, suggesting that MSC-derived secretome containing azurin elicits an anticancer effect.

Other important potential of stem cells is their application for regenerative medicine and developing new Advanced therapeutic medicinal products (ATMPs), namely for cell therapies (Samsonraj et al., 2017). In particular, human MSCs have gather special interest as a universal and feasible add-on therapy for several pathologies. This has been also extensively explored in this issue by Coelho et al., Laundos et al., and Silva M. et al.. Coelho et al., in a critical and very actual revision paper, approach MSCs as a potential therapeutic strategy in COVID-19 patients. On the other hand, Laundos et al., in a murine myocardial infarction model, show that umbilical cord matrix (UCM)-MSC based cellular products improved cardiac function and limited adverse cardiac remodeling post-ischemic injury, supporting the sustained and long-term beneficial therapeutic effect of MSCs. Silva A. C. et al., in turn, review the impact of extracellular matrix (ECM) alterations on cardiac cells, throughout heart ontogeny and disease, a hot topic as well (Bonnans et al., 2014). They further debate on available strategies based on cell–ECM interactions, toward the design of new regenerative therapies. Finally, Inácio et al., further highlight the importance of deciphering the regenerative mechanisms of mammalian adult heart. Indeed, heart failure, due to cardiomyocyte loss, is still one of the significant health burdens worldwide. In order to further contribute to the field, the authors demonstrate that DAN domain family member 5 precursor (DAND5) is a key driver for the generation and expansion of iPSC-derived cardiomyocytes systems, and therefore with further clinical application purposes.

We are particularly pleased to present this Collection and hope our readers will consider this a useful resource for the state of the art in the emerging field of stem cells and cell therapies. We also thank all contributing authors, referees and Frontiers journals.

## Author Contributions

JPM and SS wrote the Editorial based on their critical analysis of all articles submitted in the special issue on the 11^th^ Edition of the International Meeting of the SPCE-TC: Advances in Stem Cells and Cell Therapies. Both authors contributed to the article and approved the submitted version.

## Conflict of Interest

The authors declare that the research was conducted in the absence of any commercial or financial relationships that could be construed as a potential conflict of interest.
